# Documenting the experiences of health workers expected to implement guidelines during an intervention study in Kenyan hospitals

**DOI:** 10.1186/1748-5908-4-44

**Published:** 2009-07-23

**Authors:** Jacinta Nzinga, Patrick Mbindyo, Lairumbi Mbaabu, Ann Warira, Mike English

**Affiliations:** 1 KEMRI Centre for Geographic Medicine Research – Coast, KEMRI/Wellcome Trust Programme, PO Box 43640, Nairobi, Kenya; 2 Department of Paediatrics, University of Oxford, John Radcliffe Hospital, Headington, Oxford, UK

## Abstract

**Background:**

Although considerable efforts are directed at developing international guidelines to improve clinical management in low-income settings they appear to influence practice rarely. This study aimed to explore barriers to guideline implementation in the early phase of an intervention study in four district hospitals in Kenya.

**Methods:**

We developed a simple interview guide based on a simple characterisation of the intervention informed by review of major theories on barriers to uptake of guidelines. In-depth interviews, non-participatory observation, and informal discussions were then used to explore perceived barriers to guideline introduction and general improvements in paediatric and newborn care. Data were collected four to five months after in-service training in the hospitals. Data were transcribed, themes explored, and revised in two rounds of coding and analysis using NVivo 7 software, subjected to a layered analysis, reviewed, and revised after discussion with four hospital staff who acted as within-hospital facilitators.

**Results:**

A total of 29 health workers were interviewed. Ten major themes preventing guideline uptake were identified: incomplete training coverage; inadequacies in local standard setting and leadership; lack of recognition and appreciation of good work; poor communication and teamwork; organizational constraints and limited resources; counterproductive health worker norms; absence of perceived benefits linked to adoption of new practices; difficulties accepting change; lack of motivation; and conflicting attitudes and beliefs.

**Conclusion:**

While the barriers identified are broadly similar in theme to those reported from high-income settings, their specific nature often differs. For example, at an institutional level there is an almost complete lack of systems to introduce or reinforce guidelines, poor teamwork across different cadres of health worker, and failure to confront poor practice. At an individual level, lack of interest in the evidence supporting guidelines, feelings that they erode professionalism, and expectations that people should be paid to change practice threaten successful implementation.

## Introduction

Evidence-based medicine (EBM) is the conscientious, explicit, and judicious use of current best evidence in making decisions about the care of individual patients [1]. At its heart lies the logic that if the best research identifies a form of practice that improves patient or health system outcomes, then it should be adopted by health care practitioners wishing to improve patient outcomes. Evidence-based guidelines are a means by which the best evidence is aggregated to define optimal and sequential decisions in providing clinical care, for example, to a child presenting with pneumonia. Although EBM has been widely endorsed in theory, problems persist with implementation [2]. In Kenya, hospitals have not adopted World Health Organization (WHO) guidance on best practice in the care of children and newborns, although such guidance has been endorsed by the Kenyan Ministry of Health, and the care provided has previously been shown to be poor [3,4]. Therefore, we planned an intervention study aimed at improving care for seriously ill children and newborns admitted to Kenyan government district hospitals through facilitated and supervised introduction and reinforcement of best practices following training and introduction of evidence-based guidelines.

In accompanying papers or in previously published work we have described: the development of the evidence-based clinical practice guidelines (CPGs), job aides (standard medical admission record forms, guideline booklets and wall charts), and a training course based around these called Emergency Triage Assessment and Treatment plus Admission Care (ETAT+) in Kenya [5,6]; the design of a study to test the implementation of these guidelines [6]; details of the context within which the intervention is taking place[7]; and the approach to implementation that combined initial training with limited reinforcement training, supervision, feedback and local facilitation over a period of 18 months [8]. This package of interventions was felt to be appropriate and feasible in the context. The intervention package was provided to four hospitals, while a very limited intervention, comprising a dissemination seminar on the guidelines and written feedback after survey visits, was provided to four control hospitals [6].

The starting point for our work was the local rationale and evidence [6,9] supporting the intervention package design. Although there can clearly be overlap between the elements, for simplicity these were considered to comprise: training, guidelines, and the standards these imply; supervision provided by an external agency; feedback after formal evaluation; and facilitation provided by a local health worker. Again, for simplicity, we envisaged that such elements could be considered to act through a variety of possible mechanisms to help change practices and at three primary levels: at the hospital, institutional, or organizational level; at a social, team, or group level among health workers; and at an individual level. In this sense, our working approach resembles the multi-level framework for change proposed by Ferlie and Shortell[10]. In this framework, a fourth level is envisaged, the larger system or environment in which the institution is embedded. Factors at this fourth level that might affect the interventions success are described elsewhere [7], while the main aim of this report is to describe factors reported by health workers that might impede the uptake of best practices, and thus prevent improvement in the quality of care.

## Methods

### General study approach

At the onset of this study, we had a relatively simple concept of how we hoped the intervention's components might act, through a variety of mechanisms, to promote uptake of new best practices in study hospitals through influence at levels crudely characterized as: the hospital administration, hospital departments or teams, and the individual (Table 1). These initial concepts were informed by the considerable experience of some authors of working with rural Kenyan hospitals and insights from a variety of perspectives in the literature on health systems, quality improvement, guideline implementation, and behavioural research [9,11-19]. Based on these perspectives, we aimed in initial work, reported here, to focus on the uptake of the new guidelines from the perspective of those health workers expected to use them. We did not adopt a specific theoretical framework to guide data collection. Instead, we were interested in exploring, broadly, barriers to uptake or implementation of new practices experienced by health workers in their hospital contexts while we planned to explore views on supervision, feedback, and training later in the course of the 18-month intervention [8]. With these intentions, we used an in-depth case study approach in the hope of describing the range and nature of barriers encountered. Investigation was confined to the four hospitals making up the intervention arm of a comparative study. These four hospitals (H1, H2, H3, H4) are all in the government sector, and their selection and the degree to which these hospitals are representative of many other Kenyan hospitals have been discussed in detail elsewhere [6,7].

**Table 1:** Illustration of how, at the study design stage, it was considered that the four main elements of the intervention might help foster change in health care practices through effects at three main levels within hospitals.

**Level of action**	**Components of the intervention and mechanisms anticipated by the research team through which they might influence practices**			
	
	**Training, Guidelines & Standards**	**External Supervision**	**Feedback**	**Local Facilitation**
	
**Organisation** Hospital Administration, Clinical and Departmental Leadership	Clarifying technical goals, essential roles, resources and support systems required to provide best practice care Adoption and institutional ownership of standards	Evaluation against standards Encouragement and support for change Re-affirmation of guidelines and standards Promoting leadership Promotion of organizational change	Gauging success against goals Recognising and valuing positive change Promoting recognition of the 'owners of success' and local achievement Identifying continued needs and new goals Promotion of sense that 'performance matters'	Agent for addressing critical resource needs Promotion and continuous reminder of needs and goals Emissary for change
			
**Social groups** 'Culture of Practice'	Credible and authoritative new practice guidelines Creation of a critical mass to support adoption of new practice and, through peer influence, discourage non-compliance Promotion of teamwork across cadres	Re-training and strengthening skills Recognition of good performance Promoting team leadership Promotion of departmental change Support for early adopters Promote challenging of poor performance		Re-training, orientation and strengthening skills Local recognition of good performance Promoting team working Advocate and channel for communication about change Support for early adopters Local reminder/prompt
				
**Individual Practice**	Provision of knowledge and skills Availability of prompts and reminders		Reflection on personal contribution	

### Study population

Within the hospitals, health workers recruited for this study were selected based on the following criteria: health worker type – medical officer (MO), clinical officer (CO, clinicians with a three-year diploma in medicine), MO intern, CO intern, and nurses; health workers directly involved in pediatric care at the time of the visit working in the pediatric ward, the maternity unit, the out-patient department (OPD) and the maternal and child health department (MCH); administrative staff involved in implementation of new policies, such as the hospital's medical superintendent, senior nurse, senior CO, health administrative officer, and those in charge of the various pediatric departments. The hospital selected local facilitators whose selection and role is described elsewhere[8].

We used a multi-stage sampling procedure. Initially, health workers in hospitals whose duties involved working in or management of the pediatric areas at the time the investigator (JN) visited were considered eligible. Within this sample, health workers of the cadres listed above were purposively selected with the intention that this sample should include some health workers who had attended the ETAT+ training or other introduction to the guidelines. The aim of sampling was to ensure that the maximum variation in opinion might be captured, and thus continued until the point of saturation (when little new was being offered by new interviewees). The data collection was undertaken in March 2007, approximately four to five months into the 18-month intervention project whose beginning was marked by the provision of a five and one-half day training for approximately 32 staff in each of the hospitals to introduce the CPGs.

### Study tools

While development of the interview guide was aimed at a broad characterization of barriers, and not based on any specific theoretical approach, we found reports of the Theory of Planned Behaviour in research applied to health care settings [11,19,20] and the framework applying psychological theory to the field of guideline implementation developed by Michie, *et al*. [12] useful in framing questions. These models and frameworks in particular prompted exploration of aspects of self-efficacy/locus of control, beliefs about consequences that might follow use of the guidelines, and social influences or social norms in addition to exploration of basic institutional and organizational characteristics that might affect guideline uptake.

The interview guide developed was piloted at the Kenyatta National Hospital, a non-study hospital, responses were analyzed, and questions revised to develop the final interview guide for the first phase of data collection. Where appropriate, additional questions and themes were explored as new issues, originating from the interviewees, emerged in the course of the research. All the interviews were conducted in English, each lasting between 20 to 50 minutes. Additional data sources used to help interpret and analyse these data included records kept in field notes of informal discussions, and from non-participant observations made by the principal investigator (JN) during hospital visits of clinical management or hospital-organized mortality or educational meetings, where this was possible.

### Data analysis

All the interviews and field notes were transcribed by the principal researcher (JN). In the first instance, these data were then independently coded into themes felt to emerge from the data (content analysis) by two researchers (JN and AW), after which the results were compared and discussed before arriving at an agreed set of themes for coding and final analysis using NVivo 7 software (QSR International Pty Ltd 1999 to 2006). Unanticipated themes arising from the data were incorporated into a second round of coding with free nodes representing broad categories. Further nodes were then created by grouping some of the free nodes into tree nodes by making logical connections and incorporating any emerging themes. Thus, while we attempted to allow themes to emerge from the data, our prior beliefs and understanding of the literature and our simple framework describing mechanisms through which the intervention might work are likely to have influenced the final themes identified. The final stage was a layered analysis that entailed the identification of the main and then the underlying causes of reported experiences and observations.

Preliminary analyses and interpretations were then the subject of a meeting with the one local, ministry of health employed, health worker (three nurses and one CO) selected by the four hospitals from among their own staff to act as their facilitator. These four facilitators and the principal investigator (JN) met in Nairobi at the offices of the research team. In this meeting, the research team's initial formulation of the findings was presented to the facilitators who had all worked in the intervention hospitals for more than three years as Ministry of Health employees. During and after this presentation, each of the facilitators gave their accounts of, and comments on, the research team's reports from their perspective as a staff member in an intervention hospital. This discussion was used to help ensure the themes identified by our analyses made sense to those within the institutions studied.

## Results

A total of 29 health workers were interviewed across the different sites (Table 2). From the analysis, we have identified ten major themes of importance as barriers to uptake of guidelines within the first six months of our intervention.

**Table 2:** Number of participants interviewed in each hospital and cadre.

**HOSPITAL**	**H1**	**H2**	**H3**	**H4**	**TOTAL**
**Medical Officers**	1	1	2	2	6

**Clinical Officers**	4	3	2	4	13

**Clinical Officer interns**	1	1	0	0	2

**Nurses**	1	1	2	1	5

**Administrative Staff**	2	1	0	0	3

**TOTAL**	9	7	6	7	29

### Incomplete training coverage resulting in inadequate knowledge and skills

The most common response from the health workers on what barriers they faced in the implementation of guidelines was that not everyone was trained, resulting in a lack of knowledge and skills to use the guidelines among health workers in general. Although the initial training offered targeted 32 health workers per site, this still represents a modest proportion of a hospital's staff, and trained staff were often lost from pediatric areas through frequent staff internal rotations or external transfers.

### Inadequacies in standard setting and leadership

Health workers routinely seemed to place very low value on methods to set standards and disseminate guidelines locally, compounding the problem of incomplete training coverage. Particular problems seemed to be with lack of systems, such as continuous medical education (CME) or peer education offered by colleagues to orient new staff or disseminate knowledge more widely. This is compounded by the attitude that senior staff could not accept teaching from the more junior staff. Consequently, health workers who did not attend primary training were rarely made formally aware of new guidelines or standards of practice:

'If you don't know...nobody orientated us. It is probably expected that from my training this patient requires a surgical clinic, so I will send him there or this and that and I will do the necessary, but nobody comes and tells you, you learn as you go along.'

'They are our colleagues, so I am sure they think that we are not capable of training them on anything. You know like there is that kind of attitude like 'what can she tell me' maybe that is why they have looked down on the (internal) training.'

This problem may be considered one aspect of poor leadership, at least in this clinical area. More generally across all the hospitals, there was considerable variation in the role of departmental in-charges, with only a few displaying clear leadership in the implementation of the new guidelines in their respective departments even if delegated this task. Senior management in the hospitals were rarely directly involved in leading, supervising, or facilitating implementation, although they did have a role in the provision of the necessary drugs, supplies, and equipment to some degree, and in re-enforcing the authority of the facilitators:

'The Med Supt delegates to the CO in charge, and the CO in charge does not take the job seriously because I know like some of the CO's can be very problematic. So the CO in charge has been delegated, but then he becomes very protective and so what I am saying is that the Med Supt was required to come and say 'this is the way it should be' and then he puts a very strong authority...'

(Talking about senior management supervision) 'They never even come to see how we work here, to ask what challenges we encounter, they don't even come So they never come to see how we are doing, they just depend on hearsay and rumors, and may be they say we are doing good work because they have never heard complains that we are not doing the work. We need them to come here so that they can see the work that we are doing, the challenges we are facing...'.

### Lack of recognition and appreciation

A system or culture unable to appreciate and recognise work done well was also reported by health workers to be a major barrier to encouraging correct practice, not just for implementing the new guidelines. They complained that there was more emphasis on work done badly, explaining that this was a major cause of loss of morale:

'(laughs) You know, sometimes it's good to encourage your colleagues when they do well ... but many are times people only go to look for faults ... that is the most unfortunate bit such that even when one small mistake has taken place it can be blown out of proportion ... and everything else you have done is forgotten ... that's the most unfortunate bit about human beings.'

While it is not only recognition from those in positions of authority that matters to health workers its absence may reinforce the view that management doesn't care:

'The community really appreciates what we do, like the milk for the children in the ward, in ward seven, it never lacks. The administration does not; it is only there to enforce things. Unless your fellow colleagues recognize, no one else does. Sometimes they are not even aware of these things, the big bosses, they are only involved in the business side of things.'

### Poor communication and teamwork

There are, in general, few or no forums or opportunities for health workers from all the hospital's pediatric areas and all cadres to meet and discuss issues. As a result, there is little opportunity to develop any widely supported goals for pediatric care in hospitals and little self-assessment, problem identification, or problem solving at a functional, organizational level. Consequently, the teamwork among health workers in the pediatric departments is scant, and in some situations completely missing. One effect of the intervention's supervision and facilitation was a considerable improvement in cross-cadre and cross-departmental communication:

'Well, we only meet as cadres .... like you will find that there is a nurses' meeting, or a COs' meeting but for all those five years I have never seen an OPD (outpatient department) meeting ... I have never.'

'Well, sometimes she (facilitator) calls us as clinicians, then at other times she calls the nurses, and I even remember if there is a communication breakdown from up there then she will come to us and tell us that 'these people aren't doing one or two', so she has been updating us.'

Several comments also pointed to inter-cadre conflicts that may be considerable barriers to dissemination and uptake of new practices:

'Between the COs and the nurses there is even hate-love relationship over time, the CO's and the MO's have the kind of relationship that is pull and push always. So I can't call it a dream team, there is no team, we work together but there is no system of working.'

'I don't want to discuss the CO's.....simply because I do not even want to think about them ... because they are the ones who make me do more work than I am supposed to be doing ... as simple as that.'

### Organizational constraints and limited resources

Health workers describe barriers at the organizational level to include staff shortages, high staff turnover, heavy workload, frequent staff rotations, and poor workflow structure. For example, in larger hospitals with MO and CO interns staffing wards it was reported that outpatient staff had little interest in improving their own practice, often resorting to simply sending all seriously ill children to the ward for clinical admission after nothing but a cursory review. There is also a sense that things are tolerated in paediatric care that would not be tolerated in other departments. For example, at the time of one visit it was observed that CO interns were the only clinical staff available in the pediatric ward of one hospital responsible (inappropriately and illegally) for all clinical decision making. There were undoubtedly at times major resource constraints, where solutions were within the power of the hospital to address these opportunities were often not taken, for example when moving staff soon after they have received specific training:

'So I think [these] kind of changeovers are not the best. Because if you are trained in something, then you really need the chance to work on it, have experience at least two, three, four years and then move on when you are satisfied that you have done the best. It's like I have moved out of pediatrics, but I have not done the best out of my training, I am not satisfied.'

### Counterproductive health worker norms

Reports indicated that the MOs and the nurses showed greater zeal in the uptake and practice of the guidelines than COs, a cadre of Kenyan substitute doctor with a three-year basic training who are major clinical service providers in district hospitals. Reports of poor task performance among COs were not restricted to guideline implementation:

'Most of our COs are trained but even after the training, they are not practicing, they just have a funny attitude, I think they feel that they know or that they knew (laughs), I don't know.'

There was some indication that the training and guidelines empowered nurses' with knowledge and skills they did not previously have, and thus gave them confidence to take a more active role in clinical guideline implementation. However, they still reported feeling unable to correct inaccurate practice or prescriptions, and very rarely committed themselves to documenting any corrections or confronting clinicians with their mistakes. In fact, in general all cadres rarely discussed mistakes made by colleagues, reporting that they avoid unnecessary confrontations by making corrections, but not following the mistake through to its source:

'There is this one clinician in OPD who is trained, but she is just a bad one ... she sends me queer diagnoses to the ward and she is not ready to be corrected, you can't talk to her, and of course she is my boss, she is above me so there is nothing I can do.'

'But the idea of following somebody and telling them here you made a mistake ... I thought that was not right to confront someone over such small things because may be they were just tired.'

### Absence of perceived benefits linked to adoption of new practices

The aim of the guidelines is to improve care in the hope that this will improve health outcomes. Again, rationally, one would expect health workers to be supportive of such outcomes and therefore the guidelines. However, developing a sense of ownership of the guidelines was rather slow. Health workers initially regarded the programme as 'an external KEMRI affair', with supervision and local facilitation only slowly breaking down this perception. At the start, another common perception was that practicing the guidelines 'for KEMRI' should be rewarded monetarily. The expectation of financial incentives was linked to the desire for further formal ETAT+ training which potential participants expected should provide out-of-pocket attendance allowances (*per diems*). The latter challenge almost certainly reflects the long-term practice of non-governmental and governmental organizations, especially where supported by vertical programmes, of providing participants with *per diems *for attending training. Thus, although intended as reasonable compensation, such payments have unintended consequences and can be a cause of considerable disenchantment:

'They did not see the impact of the CMEs we hold within the hospital, what they wanted was to be taken outside like that one week that we went, get paid the same amount of money, and be paid certificates.'

There were some initial feelings among clinicians that the guidelines and training were rather shallow and more appropriate for rural peripheral health facilities than hospitals. However, in most hospitals the value of the guidelines and training was slowly accepted, particularly after health workers experienced the intensity of the training and after reporting improving clinical results:

'To me, that attitude was only there when we started, especially the COs who were thinking, like you said, it was too shallow, probably because they thought that was all that was there in Integrated Management of Childhood Illnesses (IMCI), they did not know there was in-patient and out-patient and that it was targeting the referrals or non-referrals. But I think the attitude is now changing, even the MOs are training for it, things are changing and, you know, even the guidelines are targeting the common, the killer diseases, and so we started where the mortality was higher.'

'Well actually what has kept me going is the results ... the changes that are brought from the management of these children in the wards.'

### Difficulties accepting change

One emerging theme was the difference in adoption of the guidelines across the different clinician age groups. Senior or older clinicians were often reported to be stuck in the patterns of previous practice, although there were also exceptions to this observation. This problem was attributed to the lack of experience of being challenged to change by new knowledge. Practices and pre-service teaching have essentially remained static over periods of many years.

Q: 'Ok. For these clinicians that are resistant yet attended the ETAT+ training, why do you think they are resistant?'

(Facilitator): 'I can't tell why but I mentioned that the ones who have been in service for long are resistant to ETAT+ and the clinicians who are in OPD, almost all of them are the older clinicians in the hospital who really do not want to listen to anyone.'

'In my opinion ... its just the usual business of 'I have been doing this thing for many years. I have treated these conditions for many years. So what do you mean by telling me a child who has diarrhea does not necessarily need antibiotics'.'

### Lack of motivation

Motivation is a critical factor influencing the performance of health workers and is discussed in much greater detail in an accompanying paper [21]. Health workers reported lack of motivation for their work generally and, by extension, for practice according to the guidelines. Contributing factors included heavy workload, lack of supplies, frequent staff rotations, staff shortages, and incompetence of some colleagues. Local institutional factors included the lack of recognition and appreciation for work done by the hospital administration or senior staff and lack of, or unfair distribution of, training opportunities at seminars or workshops that provided allowances and *per diems *(as discussed above):

'Lack of motivation is an issue, you see like a person who is trained in IMCI you stay from eight to five then you go home, the next day you ... you become a stereotyped person, you lack motivation because you cannot even run elsewhere to do ABCD to make you earn a living outside your job.'

'Sometimes when you have to resuscitate a child, and you don't have the right something at the right time, that can be demoralizing.'

'You know, even when I say motivation I do not mean we should be given money ... Ok we should be paid well, but even at the hospital level we should be recognized, you know even a certificate, even given an ward to show that we are hard working.'

### Conflicting attitudes and beliefs

A wide range of attitudes and beliefs were reported by health workers as contributors to poor guideline uptake. These included ignorance, arrogance, impatience, laxity, and lack of confidence. Self-confidence (also referred to as arrogance by interviewees), the sense that a 'well-trained' health worker does not need guidance, was often combined with a feeling that the particular guidelines being implemented were too simple, not capturing the complexity of care:

'Unless ... it's ... you see at times it looks as though you do not know what you are doing when you say very severe pneumonia or very severe disease, it does not sound ... as a clinician I should say that this is pneumonia. As I was telling you, I will not come too low to say this is severe pneumonia or very severe disease, I don't classify because I feel I know what I am doing.'

There were additional specific aspects of guideline content that were contested. These included, for example, disagreement with specific recommendations for drug dosages (Phenobarbitone, Gentamicin, and Quinine) and advice to withhold antimalarial drugs from those who were not severely ill and who had a negative malaria diagnostic test. Such lack of acceptance was despite the fact that the guidelines were based on the most up-to-date evidence [5]. Interestingly, very few health workers expressed any interest in the evidence behind the new recommendations.

While there was reluctance to accept national guidelines direct observations, especially in the outpatient areas, local pharmaceutical industry representatives were able to influence the choice of drugs so that clinicians ignored the guidelines. This was reportedly because the clinicians believed that using a 'new drug' proves their competence, and also because they sometimes accrued direct monetary benefits from this activity.

## Discussion

The approach used in this study aimed to help us understand the root causes of poor guideline adherence among health workers while they were being exposed to an intervention. Direct non-participatory observations allowed for triangulation of the data collected, but it was noted that often health workers appeared more open, relaxed, and engaged during informal chats with the researcher (JN). This – and the fact that this was not an ethnographic study, with limited amounts of time spent in these hospitals – should be kept in mind when interpreting our results and comparing them with those of other studies. Furthermore, while in developed countries investigators have employed psychological theories, such as the theory of planned behavior and/or social cognitive theory, to understand uptake of guidelines and show that attitudinal and control beliefs are important predictors of health workers' intentions and actions [22-24], our ability to explore these areas was limited. Thus, we are unable to contribute to more general conceptual thinking from these disciplinary vantage points, in part due to the difficulty accessing relevant expertise when based in a low-income setting.

However, we feel the major contribution of this study is the inclusive description of the perceptions and experiences of MOs, COs, nurses, and hospital administrators in implementing new pediatric guidelines in a Kenyan hospital setting. The findings from this study indicate that the barriers to changing practice exist at multiple levels – the individual, the social, and the organizational level – and are multi-faceted and inter-linked. The barriers identified in this study are consistent with those in the literature [2,24-26]. In particular, many of the themes identified resonated with those defined as useful for investigating implementation by Michie, *et al*., including: knowledge and skills, self-standards encompassing professional identity, beliefs about capabilities, beliefs about consequences (outcomes), motivation and goals, environmental constraints, social influences and nature of the behaviours (breaking habits) [12].

However, there were also differences. These included: differences in uptake of guidelines across the different cadres of health workers, lack of demand for evidence behind new policies and guidelines, pronounced human and material resource constraints in the hospitals, and poor health worker expectations related to the desire for payment (*per diems*) to promote implementation. These are not commonly reported from high-income settings. Although the work was conducted in Kenya, we believe many of these barriers may be common to other low-income country hospital settings. Interestingly, while making guidelines simple and specific is recommended [27], we found that this runs the risk that some clinicians will feel the approach is 'too simple', perhaps because it seems to undermine their academic profession. Similarly, an explicit link between guidelines and the evidence behind them is reported to be important in their acceptance [28] in developed country settings, but was not clearly apparent in our study. This perhaps reflects a basic lack of routine exposure to any form of evidence in Kenyan district hospital settings. The reports that COs were particularly reluctant to accept change are worrying given the reliance placed on them as substitute clinicians in Kenya, although this may be confounded by the fact that they are often older than doctors in rural areas. It is an area that perhaps warrants further investigation however, given the global interest in substitute workers.

Understanding the complex interplay between environment or context, social influence, and workplace culture, individuals' personal attitudes and beliefs are considered critical in negotiating change in health systems [10], but have rarely been explored in low-income settings. The developing countries studies that have been done have often focused largely on primary care and on personal, structural, or organizational factors that influence practice [29-31]. Other relevant studies in low-income country settings have focused on health worker performance, satisfaction, and motivation [31-33], and more recently 'mindlines'[26]. Our data, we feel, indicate the importance of considering implementation at a number of levels simultaneously [10,34]. Findings suggest that hospitals are often characterized by poor organizational coordination, in both clinical and administrative areas, with few or no routine organizational structures and processes to facilitate implementation of guidelines. A clear example is the lack of a system that introduces and orients new staff to routine/standard practice. This, combined with staff deployments that seem to take little account of training received, can over time erode any institutional memory built up around specific training or guidelines. Such institutional inattention clearly threatens the correct use of guidelines [25]. Of concern, it is also clear that mistakes or failure to follow guidelines often are tolerated and ignored by all cadres – apparently to avoid confrontation with colleagues – with a failure to use such episodes as learning opportunities.

## Conclusion

For several decades, international bodies such as WHO and national governments have produced guidance on expected best practices. However, there appears to have been almost no consideration given to implementation of best practice other than the provision of printed materials and training courses that are well known to achieve little by themselves. Despite 'improving health systems' being a common current mantra, how this is actually to be achieved is rarely articulated in terms of practical approaches. Our findings and wider experience suggest that some apparently simple interventions that may help include: establishing accepted and realistic standards of care at facility levels (including orienting new staff to standards); a clear indication that reaching standards is valued using mechanisms such as supervision and recognition; identification, recognition (including promotion), and delegation of authority to practice leaders; developing team-based management and non-confrontational means of addressing errors and non-performers; and identification and elimination of critical resource 'bottlenecks'. Learning how to implement and optimize changes and future research might benefit from the disciplines of organizational management as well as behavioural sciences. Unfortunately capacity in Africa in such research areas is very limited.

Rural Kenyan hospitals are complex, are likely to be similar to those in many African settings, and our understanding of them is currently at the 'blank sheet' stage. A focused, multi-disciplinary approach might usefully benefit thousands of current health workers and millions of patients by filling this blank sheet with a radical redesign.

## Competing interests

The authors declare that they have no competing interests.

## Authors' contributions

The idea for the study was conceived by ME who obtained the funding for this project. Preparation for and conduct of the study was undertaken by all authors. JN undertook all the interviews, and with AW undertook the qualitative analysis supported by PM and LM. JN produced the first-draft manuscript to which all authors contributed during its development before ME produced the final draft. All authors approved the final version of the report.
